# Facile Preparation of MCM-41/Ag_2_O Nanomaterials with High Iodide-Removal Efficiency

**DOI:** 10.3390/nano12203678

**Published:** 2022-10-20

**Authors:** Wenlin Yu, Qinpeng Dong, Wenbin Yu, Quan Wan, Xiuli Chen

**Affiliations:** 1 Key Laboratory of Nonferrous Materials and New Processing Technology, Ministry of Education, School of Materials Science and Engineering, Guilin University of Technology, Guilin 541004, China; 2 State Key Laboratory of Ore Deposit Geochemistry, Institute of Geochemistry, Chinese Academy of Sciences, Guiyang 550081, China; 3 University of Chinese Academy of Sciences, Beijing 100049, China; 4 CAS Center for Excellence in Comparative Planetology, Hefei 230026, China

**Keywords:** MCM-41, Ag_2_O nanoparticles, nanomaterials, adsorption, radionuclides, iodide

## Abstract

The elimination of iodide (I^−^) from water is a tough subject due to its low adsorption tendency and high mobility. In this work, MCM-41/Ag_2_O nanomaterials were prepared, characterized, and employed to adsorb I^−^ from water. The Ag_2_O nanoparticles were dispersed homogeneously in the pores or at the surface of the MCM-41 support, and the Ag_2_O nanoparticles in the pores had small particles sizes due to the confinement of the mesoporous channel. The prepared MCM-41/Ag_2_O nanomaterials exhibited a higher specific surface area than previously reported Ag_2_O-based composites. The adsorption of I^−^ by the nanomaterials was able to reach equilibrium at 180 min. The MCM-41/Ag_2_O nanomaterials showed a better adsorption capacity per unit mass of Ag_2_O than pure Ag_2_O nanoparticles and previously reported Ag_2_O-based composites prepared using other supports. Furthermore, the MCM-41/Ag_2_O nanomaterials exhibited high selectivity for I^−^ in the presence of high concentrations of competitive anions, such as Cl^−^ or Br^−^, and could function in a wide range of pH. The chemical reaction between Ag_2_O and I^−^ and the surface adsorption were the main adsorption mechanisms. These results indicate that MCM-41/Ag_2_O nanomaterials are a promising and efficient adsorbent material suitable for the removal of I^−^ for practical application.

## 1. Introduction

Silver(I) oxide (Ag_2_O) nanoparticles have attracted increasing research interest in the field of environmental remediation because of their high catalytic/adsorption activities. As a semiconductor with a narrow band gap of approximately 1.2 eV, Ag_2_O has been found to be an excellent photocatalyst for the degradation of organic contaminants and water splitting under visible light [1,2]. Good adsorption activities of Ag_2_O nanoparticles for many dyes and inorganic ions have also been reported in the literature [3,4,5,6]. Ag_2_O nanoparticles have received especially intensive research attention for the elimination of radioactive iodine because of the strong affinity between silver and iodine [7,8,9,10]. As a by-product of uranium fission, radioactive iodine is of greatest environmental concern because of its low adsorption tendency, high mobility, and potential harm to humans [11,12,13]. It is well known that exposure to even minor quantities of radioactive iodine can result in an increase in mental retardation, metabolic disorders, and thyroid cancer in humans [14].

However, directly applying Ag_2_O nanoparticles for the adsorption of radioactive iodine is unfeasible because of problems with the separation and recovery of nanoparticles, and more importantly, because of the coaggregation problem, which decreases the effective surface area of nanoparticles, and thus, reduces their reaction activities. Supporting Ag_2_O nanoparticles on porous carriers, such as TiO_2_ spheres [7], chitin nanofibers [6], titanate nanolamina [15], and halloysite nanotubes [16,17] has been found to be effective in preventing coaggregation. The resulting Ag_2_O nanocomposites showed better performance in the capture of iodide or iodine vapor than the pure Ag_2_O nanoparticles. For the reported Ag_2_O nanocomposites, the particle dimension of Ag_2_O nanoparticles and the specific surface area of the nanocomposites are two important factors affecting the adsorption performance. However, the classical method for the preparation of supported Ag_2_O is the direct precipitation of Ag_2_O particles on the surface of carriers through the mixing of silver nitrate and hydroxides [16,18]. Although this method is simple, it is very difficult to control the size of Ag_2_O nanoparticles due to the too-fast reaction speed [19]. In addition, for the preparation of Ag_2_O nanocomposites, it is predicted that increasing the specific surface area of the carrier is an effective way to increase that of the composite. From this point of view, it would be very interesting to synthesize Ag_2_O nanomaterials by supporting Ag_2_O nanoparticles with a controlled size on a carrier with a large specific surface area, and to discover the effect of support on the related characteristics and adsorption activities of the Ag_2_O nanoparticles.

MCM-41, one type of ordered mesoporous material, is composed of amorphous silica with uniform and tunable pores (1.6–10.0 nm) in cylindrical-channel form [20]. MCM-41 is characterized by a large specific surface area (around 1000 m^2^/g) and narrow pore size distribution, and it possesses good hydrothermal, thermal, and hydrolytic stability [20,21]. Because of these properties, great attention has been paid to MCM-41 as a support material for metal and metal oxide nanoparticles to be employed as catalysts or adsorbents [22,23,24,25]. For example, Ag nanoparticles were recently synthesized in the mesopores of MCM-41, and the Ag/MCM-41 composite exhibited a high Hg^0^ removal capacity (at 5% breakthrough, the Hg^0^ capture capacity of the Ag/MCM-41 loaded with 1 wt% silver was 6.64 mg) [26]. In this study, due to the confinement of the mesoporous channel of MCM-41, the size of the Ag nanoparticles reduced significantly (with an average diameter of 3 nm), giving the resulting Ag/MCM-41 composite good performance. However, so far, few attempts to synthesize MCM-41-supported Ag_2_O nanoparticles for iodine adsorption have been found in the literature.

In the present study, MCM-41/Ag_2_O nanomaterials were prepared via a facile method, in which the MCM-41 support was first impregnated with silver nitrate solution under vacuuming conditions to ensure the silver source entered the channel of MCM-41; then, Ag_2_O nanoparticles were precipitated inside and outside the mesopores of MCM-41. The prepared MCM-41/Ag_2_O nanomaterials were characterized systematically and used for the adsorption of radioactive iodide (I^−^) from water. How the characteristics of the nanomaterials and condition-specific parameters affect the adsorption reaction was investigated.

## 2. Materials and Methods

### 2.1. Materials and Chemicals

Purely siliceous mesoporous MCM-41 and crystalline potassium iodide (KI) were purchased from Sigma-Aldrich. ^127^I was used as a representation in the I^−^ uptake experiments because the non-radioactive isotope of iodine possesses the same adsorption properties as the radioactive ones [27,28]. AR-grade AgNO_3_, NaOH, KCl, KBr, and 68.0% HNO_3_ were bought from Sinopharm Chemical Reagent Co., Ltd. (SCRC, Shanghai, China). Deionized water with a resistivity of 18.2 MΩ·cm was used in all experiments. All the chemicals were used as received and were not further purified.

### 2.2. Preparation of MCM-41/Ag_2_O Nanomaterials

Each MCM-41/Ag_2_O nanomaterial was prepared according to the following procedure: 0.2 g MCM-41 was first dried at 120 °C overnight; the dried MCM-41 powder was transferred into a conical flask, and then, the conical flask was vacuumized for 3 h to get rid of the air from the channels of MCM-41; after that, 50 mL of AgNO_3_ solution with a concentration of 0.01 mol/L was added into the conical flask via a funnel under vacuum; after completing the addition of the AgNO_3_ solution, the conical flask was connected to the air immediately. The suspension was then stirred for 2 h to allow the silver nitrate to enter the mesoporous channels of MCM-41; then, 4 mL of a NaOH solution with different concentrations was added dropwise into the conical flask using a peristaltic pump at a 100 μL/min flow rate while vigorously stirring the suspension. After stirring for 8 h, the solid was collected, washed, and then, dried at 80 °C, to yield the MCM-41/Ag_2_O nanomaterials. The final products prepared using 0.02, 0.05, and 0.125 mol/L NaOH solutions were denoted as 2-Ag_2_O@MCM-41, 5-Ag_2_O@MCM-41, and 12.5-Ag_2_O@MCM-41, respectively. For comparative purposes, pure Ag_2_O nanoparticles (named Ag_2_O-NPS) were also prepared by mixing 50 mL of 0.01 mol/L AgNO_3_ with 4 mL of 0.05 mol/L NaOH using a similar procedure to that of the MCM-41/Ag_2_O nanomaterials.

### 2.3. Characterization Methods

The X-ray diffraction (XRD) patterns were obtained using a Panalytical Empyrean multifunction X-ray diffractometer (Panalytical, Almelo, The Netherlands) equipped with a three-dimensional (3D) PIXcel detector at the Institute of Geochemistry, Chinese Academy of Sciences. The test voltage and current were 40 kV and 40 mA, respectively. A continuous-scanning mode with a step size of 0.026° and a counting time of 30 s per step was used.

Transmission electron microscope (TEM) pictures were acquired using an FEI Tecnai G2 F20 S-TWIN TEM (FEI, Hillsboro, OR, USA) at 200 kV. The appropriate amount of MCM-41 or the MCM-41/Ag_2_O nanomaterial samples was sonicated and dispersed in ethanol medium for 5 min, and the suspension of the sample was then dropped on the Cu grid and air-dried before being transferred into the microscope for TEM observation.

The nitrogen (N_2_) adsorption–desorption isotherms were obtained at liquid-nitrogen temperature using a Quantachrome Autosorb-iQ2-MP gas adsorption analyzer (Quantachrome, Boynton Beach, FL, USA). The samples were degassed under vacuum at 200 °C for 12 h prior to measurement. The specific surface area values of the samples, *S*_BET_, were calculated from the nitrogen adsorption data using the multiple-point Brunauer–Emmett–Teller (BET) method [29], and the total pore volume, *V*_total_, was evaluated based on N_2_ adsorption capacity at a relative pressure of approximately 0.99. The pore size distribution (PSD) curves were derived using the nonlocal density functional theory (NLDFT) method (N_2_ at 77K on silica, NLDFT adsorption branch, cylindrical pore) [11].

X-ray photoelectron spectroscopy (XPS) analyses were conducted using a Thermo Fisher Scientific Escalab 250 spectrometer (Thermo Fisher Scientific Ltd., UK). The instrument was equipped with a monochromatic Al Kα source, and a measurement voltage of 1486.8 eV was used. When conducting the experiments, the spectrometer analyzer chamber had a base pressure of less than 5 × 10^−8^ mbar, and a charge neutralizer filament was used to control the sample charge. All the spectra were collected using a pass energy of 100 eV for the wide scan and 30 eV for individual elements. All binding energies were calibrated to the C1s line with a location of 284.8 eV. Quantitative analysis of all the XPS data was conducted in Thermo Avantage v5.934 software and all spectra were corrected with the Smart Background Correction.

### 2.4. Iodide Adsorption Tests

KI was dissolved in deionized water to prepare the I^−^ solution. For the kinetics experiments, 0.2 g of adsorbent was added to 200 mL of a 2 mmol/L (mM) I^−^ solution, and this mixture was powerfully shaken in a platform shaker at 200 rpm to ensure its complete mixing. The adsorption time was in the range of 5 to 1440 min. At the end of each adsorption time, 1 mL of suspension was taken and passed through a 0.22 μm PTFE filter. The I^−^ concentrations in the filtered solution were analyzed using a Dionex ICS-90 Ion Chromatography (IC) system (Dionex, Sunnyvale, CA, USA). The IC instrument was equipped with an AG23 guard and AS23 analytical column, and the eluent was a 14 mM Na_2_CO_3_/1.75 mM NaHCO_3_ solution. Batch adsorption experiments were conducted to obtain the adsorption isotherms. Typically, the adsorbent was mixed with I^−^ solution with scheduled concentration under a solid/liquid ratio of 20 mg/20 mL in a centrifuge tube of 50 mL. After an adsorption time of 1440 min, the suspensions were centrifuged and the supernatants were used for the I^−^ concentration determination in the same way as those used in the kinetics testing. The selective uptake of I^−^ by the nanocomposites was tested with the coexistence of high concentrations of Cl^−^ or Br^−^ anions. Specifically, 20 mg of adsorbent was assigned to 20 mL of an aqueous solution, where the concentration of a coexisting anion (Cl^−^ or Br^−^) was ten times that of I^−^. The effect of pH on the adsorption of I^−^ by the MCM-41/Ag_2_O nanocomposites was determined by adjusting the initial pH of the I^−^ solution to approximately 4.0−10.0, respectively, by adding 0.1 M NaOH or 0.1 M HNO_3_. All adsorption experiments were performed at room temperature (25 ± 2 °C). Blank experiments were conducted to exclude adsorption by the wall or the loss of I^−^ to volatilization.

The I^−^ adsorption capacity of the adsorbent at time *t* (min), *q*_t_ (mg/g), was calculated using the following expression:

*q*_t_ = (*C*_0_ − *C*_t_) × M/m (1)

where *C*_0_ and *C*_t_ (mmol/L, mM) are the concentrations of I^−^ in the reaction solution before and after adsorption, respectively; M is the molar mass of I^−^; and m (g) is the amount of adsorbent in 1 L of the I^−^ solution. The removal efficiency of I^−^, E(%), was calculated according to the following equation:

E(%) = [(*C*_0_ − *C*_t_)/*C*_0_] × 100 (2)

## 3. Results and Discussion

### 3.1. Characterization of Samples

**Figure 1:**
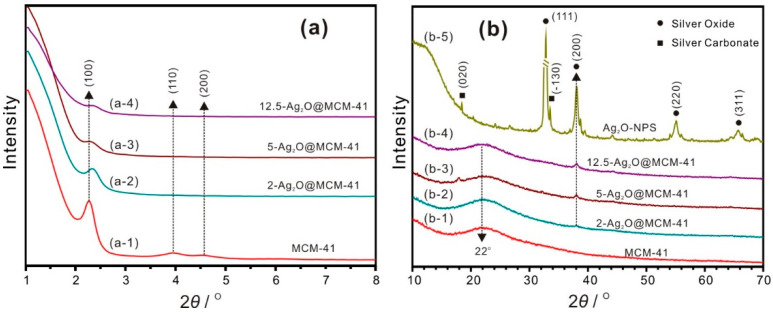
(**a**) small-angle XRD patterns and (**b**) wide-angle XRD patterns of MCM-41, Ag_2_O@MCM-41 nanomaterials with different loading concentrations, and Ag_2_O nanoparticles.

**Figure 2:**
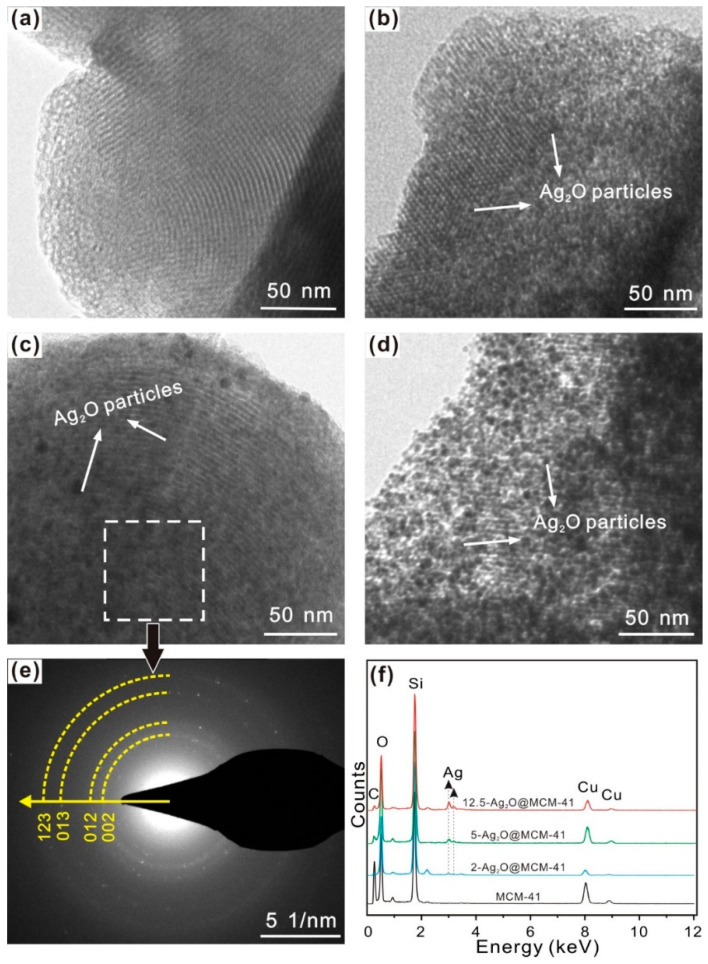
TEM images of (**a**) MCM-41, (**b**) 2-Ag_2_O@MCM-41, (**c**) 5-Ag_2_O@MCM-41, and (**d**) 12.5-Ag_2_O@MCM-41. (**e**) SAED pattern of the dashed area in (**c**), and (**f**) the EDS spectra of the prepared nanomaterials. All the spectra were normalized by peak intensities of Si element.

**Figure 3:**
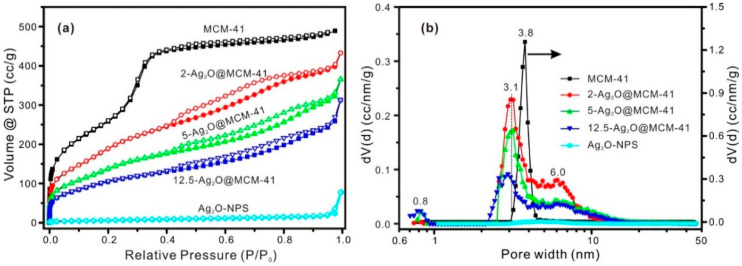
(**a**) N_2_ adsorption-desorption isotherms and (**b**) PSD curves of the MCM-41 support, the Ag_2_O@MCM-41 nanomaterials (for MCM-41, the *y*-axis is on the right; for the other samples, the *y*-axis is on the left), and the Ag_2_O-NPS.

**Figure 4:**
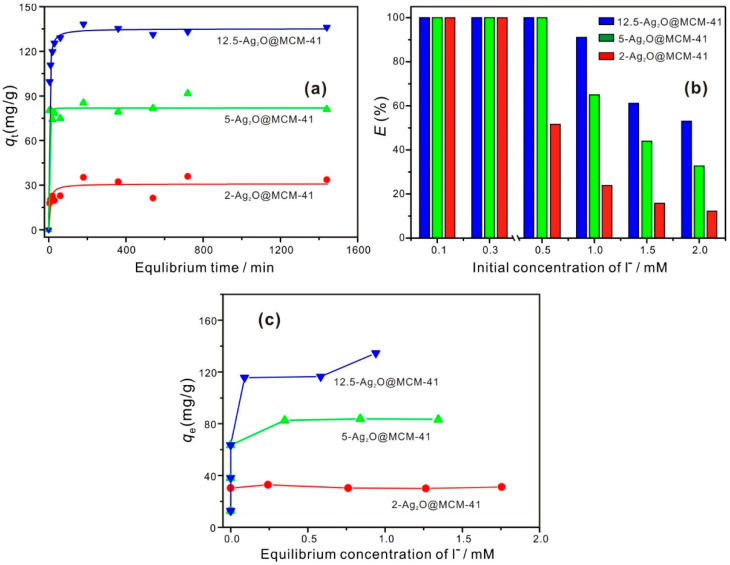
(**a**) The adsorption kinetic curves of I^−^ on the Ag_2_O@MCM-41 nanomaterials; (**b**) the removal efficiency (E) of I^−^ at different initial concentrations by the Ag_2_O@MCM-41 nanomaterials; (**c**) adsorption isotherms of I^−^ on the Ag_2_O@MCM-41 nanomaterials.

The small-angle XRD (SAXRD) patterns of the MCM-41 support and the Ag_2_O@MCM-41 nanomaterials are displayed in Figure 1a. The SAXRD pattern of MCM-41 shows three peaks at 2.3°, 3.9°, and 4.6° (2θ) (Figure 1a-1), attributed to the (100), (110), and (200) reflections of the two-dimensional hexagonal structure, respectively [20]. This result indicates the highly ordered and hexagonal mesoporous structure of MCM-41. The loading of Ag_2_O nanoparticles had a significant effect on the pore structure of MCM-41. The peak intensities of (100), (110), and (200) diffractions decreased obviously with the increasing concentrations of NaOH solution used in the preparation of the nanomaterials (Figure 1a-(2–4)). This result could be attributed to the decline in the long-range order of the hexagonal arrangement of the mesoporous structure, which was caused by blocking of the mesopores due to the increased loading amounts of Ag_2_O nanoparticles [30]. The slight shifting of the (100) reflections to the higher 2θ value for the nanomaterials (Figure 1a-(2–4)) can be explained by the fact that the pore structure of MCM-41 was slightly deformed after loading with Ag_2_O nanoparticles.

**Figure 5:**
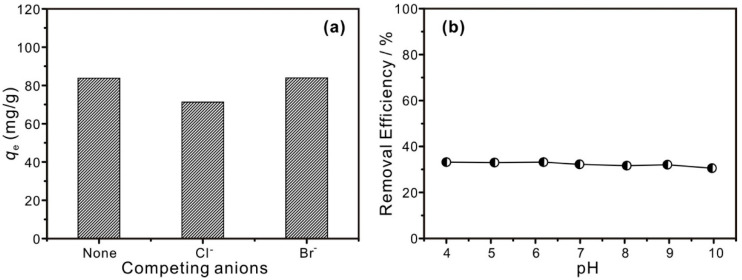
(**a**) I^−^ adsorption capacities of 5-Ag_2_O@MCM-41 under competitive adsorption conditions; (**b**) the effect of initial pH on the I^−^ adsorption on 5-Ag_2_O@MCM-41.

Figure 1b shows the wide-angle XRD patterns of the samples. The characteristic broad peaks centered at 22° for MCM-41 support and all the nanomaterials (Figure 1b-(1–4)) are indicative of the noncrystalline structure of MCM-41 [31,32]. The XRD pattern of the Ag_2_O-NPS appears in Figure 1b-5, and its main phase is silver oxide with a cubic structure, as indicated by the (111), (200), (220), and (311) reflections at 32.8°, 38.1°, 54.9°, and 65.4°, respectively. A negligible amount of silver carbonate impurity was also observed in the Ag_2_O-NPS, as indicated by Figure 1b-5, which could be explained by the effect of carbon dioxide in the air because of the slow addition rate of NaOH. A similar result was reported for the preparation of Ag_2_O using a wet chemical route whereby the pH was not controlled [33]. As seen in Figure 1b-(2–4), a new peak at 38.1° (2θ), attributed to the (200) reflection of silver oxide, appeared in the XRD patterns of all the nanomaterials, and its intensity increased in the order of 2-Ag_2_O@MCM-41, 5-Ag_2_O@MCM-41, and 12.5-Ag_2_O@MCM-41. These results indicate that silver oxide was present in the Ag_2_O@MCM-41 nanomaterials and its loading amounts increased with the concentrations of NaOH solution used in the preparation of the nanomaterials. Interesting, normally, the strongest diffraction peak for pure Ag_2_O is (111) diffraction (Figure 1b-5), but only the (200) diffraction peaks were identified for all the nanomaterials, indicating which is the strongest diffraction peak of Ag_2_O in the nanomaterials. The highly intense of (200) diffraction could be attributed to the high orientation of {100} crystal planes of the Ag_2_O nanoparticles [34] because most of them lie in the channel of MCM-41 and should be parallel to the channel.

The TEM image shows that the MCM-41 support possessed a typically ordered mesoporous structure (Figure 2a). For the Ag_2_O@MCM-41 nanomaterials, the Ag_2_O nanoparticles were dispersed homogeneously in the pores or at the surface of the support. For the sample 2-Ag_2_O@MCM-41, it seemed that most Ag_2_O nanoparticles were in the mesoporous channels of MCM-41 (Figure 2b). The amount of Ag_2_O nanoparticles in the nanomaterials increased in the order: 2-Ag_2_O@MCM-41 < 5-Ag_2_O@MCM-41 < 12.5-Ag_2_O@MCM-41 (Figure 2b–d). For the sample 12.5-Ag_2_O@MCM-41, an appreciable number of Ag_2_O nanoparticles could be found at the surface of MCM-41 (Figure 2d). Although the size of the Ag_2_O nanoparticles increased with its amount, it seemed that the size of Ag_2_O was smaller than that of the composites in which Ag_2_O nanoparticles were only at the surface of the supports [6,16]. For example, in the reported nanofibrillated chitin/Ag_2_O aerogels, the size of Ag_2_O could easily reach a diameter of more than 15 nm [6]. The circular streaking in the SAED pattern (Figure 2e) indicates that crystalline Ag_2_O nanoparticles existed in the nanomaterials. The EDS results confirmed the presence of the Ag element in the nanomaterials, whose amount increased in the order of 2-Ag_2_O@MCM-41, 5-Ag_2_O@MCM-41, and 12.5-Ag_2_O@MCM-41 (Figure 2f). The Ag_2_O loading amounts in the nanomaterials calculated using the Ag content from the EDS are listed in Table 1. Figure S1 shows the TEM images of pure Ag_2_O nanoparticles, which tended to aggregate dramatically. This result indicates that the preparation of Ag_2_O@MCM-41 nanomaterials inhibited the aggregation of the Ag_2_O nanoparticles.

Figure 3 presents the N_2_ adsorption-desorption isotherms and the PSD curves of both the MCM-41 support and the relative nanomaterials. The textural parameters of different samples are listed in Table 1. According to the IUPAC classification, MCM-41 exhibited a typical adsorption isotherm of type IV(b) (Figure 3a), which is characteristic of materials with relatively narrow mesopore size distributions [35]. The PSD curve of MCM-41 revealed that the pore size of its mesoporous channel was 3.8 nm (Figure 3b). The BET-specific surface area of MCM-41 was 951.0 m^2^/g, which is significantly larger than that of other carriers used to prepare Ag_2_O-based nanocomposites (such as TiO_2_ spheres [7], chitin nanofibers [6], titanate nanolamina [15], and halloysite nanotubes [16]). The total pore volume of MCM-41 was 0.7568 cm^3^/g (Table 1).

The N_2_ adsorption-desorption isotherms for all the nanomaterials can be designated as type IV(a) with H3 hysteresis loops [35], and the N_2_ adsorption amount in the whole *P/P*_0_ range decreased significantly when compared with the MCM-41 support (Figure 3a); this result indicates diminishment of the pore structure in the nanomaterials. The PSD curves of the nanomaterials revealed three distinct pore populations centered at ca. 0.8, 3.1, and 6.0 nm, respectively (Figure 3b). The dominating one, centered at ca. 3.1 nm, is ascribed to the mesoporous channel of MCM-41 in which the Ag_2_O nanoparticles were dispersed, and this decrease in the mesopore size from 3.8 nm is a clear indication of the Ag_2_O nanoparticles produced in the mesoporous channel. As indicated in Figure 3b, the intensity and position of the pore populations ascribed to these mesoporous channels were related to the content of Ag_2_O in the nanomaterials. The other pore populations (centered at ca. 0.8 and 6.0 nm) likely arose from the interval space or stacking of Ag_2_O nanoparticles inside or outside the mesoporous channels. The *S*_BET_ and *V*_Total_ values of the nanomaterials were lower than those of the MCM-41 support (Table 1), due to the filling or blocking of the mesopores by Ag_2_O nanoparticles. However, it can be found that the *S*_BET_ values of the nanomaterials prepared in this study were much larger than those of other reported Ag_2_O-based nanocomposites [6,15,16]. In addition, the cumulative pore volume of pores of <3.8 nm (*V*_pore<3.8nm_) of different samples was also calculated and the results are listed in Table 1. The *V*_pore<3.8nm_ of the nanomaterials was also lower than that of MCM-41, indicating that some Ag_2_O nanoparticles existed inside the mesoporous channel of MCM-41 in the nanomaterials.

The isotherm of the Ag_2_O-NPS is best characterized as being a type II isotherm, and the sharp increase near the relative pressure of 1.0 in the adsorbed amount of N_2_ corresponds to the adsorption by macropores, which were formed by the stacking or the interval space of Ag_2_O nanoparticles (Figure S1). The PSD curve shows no obvious microporous or mesoporous populations of Ag_2_O-NPS (Figure 3b). The low *S*_BET_ value of the Ag_2_O-NPS (Table 1) was due to the dramatic aggregation of Ag_2_O nanoparticles and the high density of Ag_2_O [17] (Figure S1).

### 3.2. Iodide Adsorption Performance of Samples

To establish the equilibrium time and determine the kinetics of the adsorption process, the adsorption of I^−^ on the Ag_2_O@MCM-41 nanomaterials was studied as a function of contact time. As shown by Figure 4a, the adsorption of I^−^ by the nanomaterials can reach equilibrium at 180 min, which is faster than the equilibrium time of the adsorption of I^−^ by minerals, such as illite and chrysotile [17,36]. This rapid adsorption may be related to the adsorption mechanism involved in the present study is based on a chemical reaction between Ag_2_O and I^−^. In addition, the good dispersion and the small size of Ag_2_O particles in the nanomaterials could also contribute to the rapid adsorption of I^−^, because they are readily accessible to I^−^ compared to Ag_2_O agglomerates or large Ag_2_O particles. The kinetic data were fitted using different models, such as pseudo first order, pseudo second order, simplified elovich, and Weber and Morris models [37], and the pseudo-second-order kinetic model demonstrated the best fit in quantitatively describing the adsorption data (Table S1). This result implied that the rate-limiting step for I^−^ adsorption by the prepared nanomaterials may be chemical adsorption [38]. To ensure adsorption equilibrium, a contact time of 1440 min was used for the following adsorption test.

Figure 4b shows the effect of initial concentrations of I^−^ on the adsorption performance of the nanomaterials. As indicated in Figure 4b, below 0.3 mM, all three nanomaterials could remove 100% of I^−^ from the solution, and the removal efficiency of I^−^ was 23.9%, 65.0%, and 91.0% for 2-Ag_2_O@MCM-41, 5-Ag_2_O@MCM-41, and 12.5-Ag_2_O@MCM-41, respectively, when the initial concentration increased to 1.0 mM. These results indicate that the I^−^ removal efficiency of the nanomaterials was related to the contents of Ag_2_O in the nanomaterials.

The adsorption isotherm results (Figure 4c) revealed that the maximum adsorption capacity (*q*_m_) of the nanomaterials were in the following order: 2-Ag_2_O@MCM-41, 31.1 mg/g; 5-Ag_2_O@MCM-41, 83.2 mg/g; and 12.5-Ag_2_O@MCM-41, 134.6 mg/g. As a control, the MCM-41 support was also used to adsorb I^−^, and negligible adsorption was found for MCM-41, with a maximum adsorption capacity of 0.3 mg/g. These results indicate that the adsorption capacity of the nanomaterials mainly comes from the Ag_2_O nanoparticles. As shown in Table 2, the *q*_m_ values of the Ag_2_O@MCM-41 prepared in this study were compared with other Ag_2_O-based composites prepared using different supports. The *q*_m_ values of the Ag_2_O@MCM-41 nanomaterials were lower than those for other Ag_2_O-based composites, such as Ag_2_O/titanate nanolamina [15], Ag_2_O/Titanate nanofibers [18], and Ag_2_O@ChNF aerogels [6], which is due to the lower Ag_2_O contents in our nanomaterials (Table 2). For example, the Ag_2_O content in the Ag_2_O@ChNF aerogels is as high as 31.00 wt.% [6], but the maximum Ag_2_O content in our nanocomposites is only 8.94 wt.%. In addition, the *q*_m_ values of the Ag_2_O@MCM-41 nanomaterials were also compared with other materials and the results are listed in Table S2. It can be found that the *q*_m_ values of the Ag_2_O@MCM-41 nanomaterials are considerably higher than those for black carbon [14], chrysotile [11], and layered double hydroxides (Mg/Al LDH) [39]. These results indicate that the nanomaterials prepared in this study have the potential to be an efficient adsorbent for the removal of I^−^.

To compare the I^−^ adsorption efficiency of different nanomaterials, the maximum adsorption capacity normalized to the per-unit mass of Ag_2_O content, *q*_m-Ag_2_O_, was calculated, as listed in Table 2. It was found that the *q*_m-Ag_2_O_ values of the Ag_2_O@MCM-41 nanomaterials were significantly higher than those of the other Ag_2_O-based composites. The following reasons can explain this result: (1) the MCM-41 support has a larger specific surface area than that of the other supports, so the Ag_2_O particles supported on MCM-41 exhibited better dispersion than those on the others; (2) the particle sizes of Ag_2_O in Ag_2_O@MCM-41 nanomaterials were smaller than those in the other composites because a portion of Ag_2_O nanoparticles resided in the mesoporous channel of MCM-41, which cannot grow to a big size due to the confinement of the channel. In addition, the *q*_m-Ag_2_O_ values of the Ag_2_O@MCM-41 nanomaterials were also higher than that of pure Ag_2_O-NPS, due to the better dispersion of Ag_2_O nanoparticles than their homogeneous counterparts. These results indicate that supporting Ag_2_O particles in MCM-41 can increase their efficiency for the adsorption of I^−^.

The selectivity of the Ag_2_O@MCM-41 nanomaterials in removing I^−^ was investigated when I^−^ coexisted with a high-concentration competing anion (Cl^−^ or Br^−^). As displayed in Figure 5a, the I^−^ adsorption capacities of 5-Ag_2_O@MCM-41 were hardly affected by the coexistence of the competing anion, even if the concentration of Cl^−^ or Br^−^ was ten times that of I^−^. This result suggests that the prepared nanomaterials possess a high selectivity to I^−^. The pH shows no noticeable effect on the I^−^ removal efficiency in the range of ca. 4–10 (Figure 5b), suggesting that the prepared nanomaterials could function in a wide pH range.

### 3.3. Iodide Adsorption Mechanisms

**Figure 6:**
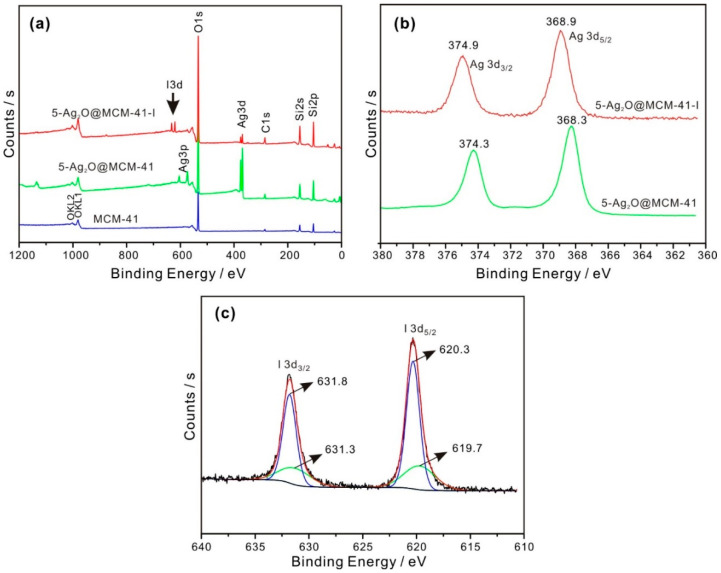
(**a**) XPS survey scan spectra of MCM-41, 5-Ag_2_O@MCM-41, and after its adsorption of I^−^; (**b**) high-resolution XPS Ag3d spectra of 5-Ag_2_O@MCM-41 before and after adsorption of I^−^; (**c**) high-resolution XPS I3d spectra of 5-Ag_2_O@MCM-41 after adsorption of I^−^ (initial I^−^ concentration, 1.5 mM; 180 min).

To study the adsorption mechanisms between I^−^ and the prepared Ag_2_O@MCM-41 nanomaterials, the solid samples were recovered after the adsorption experiments and characterized via XPS. The I^−^ adsorbed samples are indicated by the “-I” suffix. Since the adsorption mechanisms of all the prepared nanomaterials are similar, here, we take 5-Ag_2_O@MCM-41 as an example to illustrate the adsorption mechanism. As shown in Figure 6a, the I3d peaks emerged in the XPS survey scan spectra of 5-Ag_2_O@MCM-41-I, confirming that this prepared nanomaterial can uptake iodide from water.

High-resolution XPS Ag3d and I3d spectra were obtained to study the chemical status of silver and iodine. The Ag3d5/2 and Ag3d3/2 peaks of 5-Ag_2_O@MCM-41 were located at 368.3 and 374.2 eV, respectively (Figure 6b), which correspond to the typical binding energy for Ag in Ag_2_O [40]. After the adsorption of I^−^, the Ag3d5/2 peak of 5-Ag_2_O@MCM-41-I underwent a positive shift with central binding energy at 368.9 eV (Figure 6b). This shift indicates the change in the chemical environment of Ag after the adsorption of I^−^, which may be related to the form of AgI. Regarding this chemical shift, it is noteworthy that a negative shift in Ag binding energy was also previously reported by us [17] and another researcher [18] based on the lower electronegativity of iodine than oxygen. While we are currently unable to explain this inconsistence, it must be related to the specificity of Ag, since an opposite direction for the chemical shift was previously reported for the Ag, Ag_2_O, and AgO systems compared to all other metal–metal oxide systems [41]. 

The high-resolution I3d spectra of 5-Ag_2_O@MCM-41-I along with their fitting results are presented in Figure 6c. The I3d5/2 peak of 5-Ag_2_O@MCM-41-I were deconvoluted into two components occurring at 619.7 eV and 620.3 eV. The signal at 619.7eV was attributed to I^−^ in AgI [42], while the peak at 620.3 eV could be ascribed to I_2_ [43], which was formed due to the partial oxidation of iodide by the dissolved oxygen. Li et al. and Mao et al. have, respectively, reported that iodide could be partially oxidized by dissolved oxygen, forming I_2_ [42,44]. The generated I_2_ can be adsorbed by AgI at the surface of the prepared nanomaterials. Accordingly, we surmised that the iodide adsorption mechanism is based on the reactions below:

Ag_2_O + 2I^−^ + H_2_O → 2AgI + 2OH^−^ (3)

2I^−^ + O_2_ → I_2_ + 2O^2^^−^ (4)

AgI + I_2_ → AgI_3_ (5)

The reactions ((4) and (5)) that should occur during the adsorption process can be further confirmed by the high adsorption capacities of per unit mass of Ag_2_O in the nanomaterials (Table 2). If reaction (3) was the only adsorption mechanism, the theoretical maximum I^−^ adsorption capacity of Ag_2_O should be 1095.8 mg/g [17]. As seen in Table 2, for all the nanocomposites, the adsorption capacities normalized to the per-unit mass of Ag_2_O content, *q*_e-Ag_2_O_, were larger than the theoretical maximum I^−^ adsorption capacity of Ag_2_O. These results indicate that surface adsorption between the formed AgI and I_2_ may occur. In addition, some I^−^ could also be adsorbed at the surface or in the nanopores of the nanomaterials due to their high specific surface area and pore volume.

## 4. Conclusions

In summary, MCM-41/Ag_2_O nanomaterials with different Ag_2_O contents were prepared via a facile method. The nanomaterials were characterized using a series of techniques and used to remove I^−^ from water. The Ag_2_O nanoparticles dispersed homogeneously in the pores or at the surface of the MCM-41 support. Due to the highly specific surface area of the support and the small size of Ag_2_O nanoparticles, the prepared MCM-41/Ag_2_O nanomaterials exhibited a higher specific surface area than previously reported Ag_2_O-based composites. The adsorption of I^−^ by the nanomaterials were able to reach equilibrium at 180 min. The MCM-41/Ag_2_O nanomaterials showed a better adsorption capacity per unit mass of Ag_2_O than pure Ag_2_O nanoparticles and previously reported Ag_2_O-based composites prepared using other supports. Furthermore, the MCM-41/Ag_2_O nanomaterials exhibited high selectivity for I^−^ with the coexistence of high concentrations of Cl^−^ or Br^−^, and could function in a wide pH range. These results indicate that Ag_2_O@MCM-41 nanomaterials could be a promising adsorbent for the efficient removal of I^−^ for practical application, but further investigations concerning the adsorption of wastewater from nuclear-accident sites or polluted groundwater need to be performed.

## Figures and Tables

**Table 1:** The Ag_2_O contents and the textural parameters of different samples.

Samples	*W*_Ag_ (wt.%) ^1^	*W*_Ag_2_O_ (wt.%) ^2^	*S*_BET_ (m^2^/g)	*V*_Total_ (cm^3^/g)	*V_pore_*_<3.8 nm_ (cm^3^/g)
MCM-41	--	--	951.0	0.7568	0.4674
2-Ag_2_O@MCM-41	0.87	0.93	724.8	0.6157	0.2102
5-Ag_2_O@MCM-41	4.59	4.93	520.6	0.4985	0.1553
12.5-Ag_2_O@MCM-41	8.32	8.94	395.1	0.4000	0.1040
Ag_2_O-NPS	--	--	22.7	0.0367	0.0018

^1^ *W*_Ag_, Ag content in the nanomaterials, determined by the EDS results, which are the average values of the EDS data. ^2^ *W*_Ag_2_O_, Ag_2_O content in the nanomaterials, which was calculated from the *W*_Ag_ using the following equation: *W*_Ag_2_O_ = *W*_Ag_ × (107.9 + 8)/107.9.

**Table 2:** Comparison of I^−^ adsorption capacities on various Ag_2_O-based composites.

Composites	Supports	*q*_m_ (mg/g)	*W*_Ag_2_O_ (wt. %) ^1^	*q*_m-Ag_2_O_ (mg/g) ^2^	Ref.
2-Ag_2_O@MCM-41	MCM-41	31.1	0.93	3344.1	This work
5-Ag_2_O@MCM-41	MCM-41	83.2	4.93	1687.6	This work
12.5-Ag_2_O@MCM-41	MCM-41	134.6	8.94	1505.6	This work
Ag_2_O/halloysite	Halloysite	57.7	6.36	907.2	[16]
Ag_2_O/titanate nanolamina	Titanate nanolamina	431.8	43.29	997.5	[15]
Ag_2_O/titanate nanofibers	Titanate nanofibers	381.0	Not given	--	[18]
Ag_2_O@ChNF aerogels	Chitin-based aerogels	~304.8	31.00	983.2	[6]
Ag_2_O-NPS	--	743.9	100.00	743.9	This work

^1^ *W*_Ag_2_O_, Ag_2_O content in the nanomaterials. ^2^ *q*_m-Ag_2_O_, the maximum adsorption capacity of adsorbents normalized to per-unit mass of Ag_2_O, which was calculated according to *q*_m_ and Ag_2_O content in the composites using the following equation: *q*_m-Ag_2_O_ = *q*_m_/*W*_Ag_2_O_, where I^−^ adsorption of the corresponding supports is very minor related to that of Ag_2_O particles, so that is overlooked here.

## Data Availability

The data presented in this study are available on request from the corresponding author.

## Supplementary Materials

The following supporting information can be downloaded at: https://www.mdpi.com/article/10.3390/nano12203678/s1, Figure S1: (a,b) TEM images of pure Ag_2_O nanoparticles, (c) HR-TEM image of a Ag_2_O nanoparticles, and (d) its integrated pixel intensities; Table S1: Kinetics constants for I^−^ adsorption in various samples; Table S2: Comparison of adsorption capacities of different adsorbents for I^−^. References [11,14,16,39,45,46,47] are cited in the Supplementary Materials.
