# Supplementary material for: Facile Preparation of MCM-41/Ag2O Nanomaterials with High Iodide-Removal Efficiency
Source: Nanomaterials (Basel). 2022 Oct 20;12(20):3678. doi: 10.3390/nano12203678 (PMC9610164; doi:10.3390/nano12203678)
Supplement: Supplementary file 1 [file nanomaterials-12-03678-s001.zip › nanomaterials-1966804-supplementary.pdf]

Supplementary Data

Facile Preparation of MCM-41/Ag_2O Nanomaterials with High Iodide-Removal Efficiency

Wenlin Yu [1,2], Qinpeng Dong [1,2], Wenbin Yu [2,3,*], Quan Wan [2,3,4] and Xiuli Chen [1,*]

[1] Key Laboratory of Nonferrous Materials and New Processing Technology, Ministry of Education, School of Materials Science and Engineering, Guilin University of Technology, Guilin 541004, China
[2] State Key Laboratory of Ore Deposit Geochemistry, Institute of Geochemistry, Chinese Academy of Sciences, Guiyang 550081, China
[3] University of Chinese Academy of Sciences, Beijing 100049, China
[4] CAS Center for Excellence in Comparative Planetology, Hefei 230026, China
* Correspondence: yuwenbin@mail.gyig.ac.cn (W.Y.); cxlnwpu@163.com (X.C.)

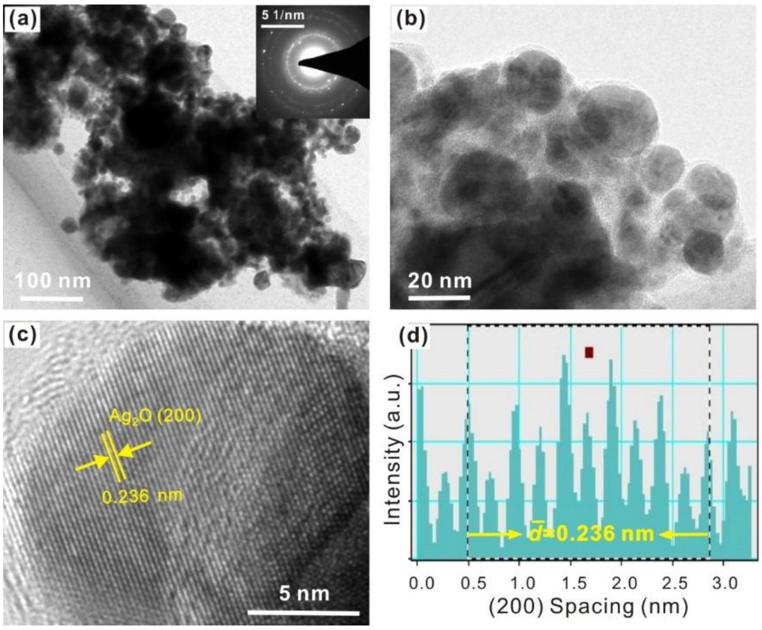

Figure. S1 (a-b) TEM images of pure Ag_2O nanoparticles, (c) HR-TEM image of a Ag_2O nanoparticles and (d) its integrated pixel intensities.

Table S1 Kinetics constants for I^- adsorption on various samples

Adsorbents	Kinetics model and related parameters											
	Pseudo-second-order model: $q_t = k_2 q_e^2 t/(1+k_2 q_e t)$			Pseudo-first-order model: $q_t = q_e(1-\exp(-k_1 t))$			Weber and Morris model: $q_t = k_d t^{1/2} + B$			Simplified Elovich model: $q_t = (1/\beta)\mathrm{Ln}(\alpha\beta t)$		
	q_e ($mg \cdot g^{-1}$)	k_2 ($g \cdot mg^{-1} \cdot min^{-1}$)	R^2	q_e ($mg \cdot g^{-1}$)	k_1 (min^{-1})	R^2	k_d ($mg \cdot g^{-1} \cdot min^{-1/2}$)	B ($mg \cdot g^{-1}$)	R^2	α ($mg \cdot g^{-1} \cdot min^{-1}$)	β ($g \cdot mg^{-1}$)	R^2
2-Ag_2O@MCM-41	30.5	0.005	0.760	28.6	0.125	0.667	0.599	15.98	0.438	305.7	0.35	0.809
5-Ag_2O@MCM-41	81.4	0.060	0.961	80.8	1.024	0.959	0.889	61.79	0.102	1.3E26	0.80	0.969
12.5-Ag_2O@MCM-41	134.6	0.0038	0.996	130.7	0.250	0.974	1.730	91.85	0.203	3.5E8	0.18	0.977

Table S2. Comparison of adsorption capacities of different adsorbents for I^-.

Adsorbents	I^- adsorption capacities	Ref.
2-Ag_2O@MCM-41	31.1 mg/g	This work
5-Ag_2O@MCM-41	83.2 mg/g	This work
12.5-Ag_2O@MCM-41	134.6 mg/g	This work
Black carbon	~2 mg/g	[1]
Activated Carbon	~12.7 mg/g	[2]
Chrysotile bundles	4.13 mg/g	[3]
Ferrihydrite	< 0.127 mg/g	[4]
Mg/Al LDH	5.8 mg/g	[5]
Halloysite/Ag_2O	57.7 mg/g	[6]
Cuprite (Cu_2O)	~0.15 mg/g	[7]

References

1. Choung, S.; Um, W.; Kim, M.; Kim, M.G. Uptake Mechanism for Iodine Species to Black Carbon. *Environmental Science & Technology* **2013**, *47*, 10349-10355, doi:10.1021/es401570a.
2. Hoskins, J.S.; Karanfil, T.; Serkiz, S.M. Removal and Sequestration of Iodide Using Silver-Impregnated Activated Carbon. *Environmental Science & Technology* **2002**, *36*, 784-789, doi:10.1021/es010972m.
3. Yu, W.; Xu, H.; Roden, E.E.; Wan, Q. Efficient adsorption of iodide from water by chrysotile bundles with wedge-shaped nanopores. *Applied Clay Science* **2019**, *183*, 105331, https://doi.org/10.1016/j.clay.2019.105331.
4. Yu, Z.; Warner, J.A.; Dahlgren, R.A.; Casey, W.H. Reactivity of iodide in volcanic soils and noncrystalline soil constituents. *Geochim. Cosmochim. Acta* **1996**, *60*, 4945-4956, https://doi.org/10.1016/S0016-7037(96)00305-5.
5. Liang, L.; Li, L. Adsorption behavior of calcined layered double hydroxides towards removal of iodide contaminants. *J. Radioanal. Nucl. Chem.* **2007**, *273*, 221-226, doi:10.1007/s10967-007-0740-x.
6. Yu, W.; Dong, Q.; Yu, W.; Qin, Z.; Nie, X.; Wan, Q.; Chen, X. Preparation of Halloysite/Ag2O Nanomaterials and Their Performance for Iodide Adsorption. *Minerals* **2022**, *12*, doi:10.3390/min12030304.
7. Lefèvre, G.; Walcarius, A.; Ehrhardt, J.J.; Bessière, J. Sorption of Iodide on Cuprite (Cu2O). *Langmuir* **2000**, *16*, 4519-4527, doi:10.1021/la9903999.
